# Treatment Outcomes in Patients With Opioid Use Disorder Who Were First Introduced to Opioids by Prescription: A Systematic Review and Meta-Analysis

**DOI:** 10.3389/fpsyt.2020.00812

**Published:** 2020-08-28

**Authors:** Nitika Sanger, Meha Bhatt, Nikhita Singhal, Balpreet Panesar, Alessia D’Elia, Maegan Trottier, Hamnah Shahid, Alannah Hillmer, Natasha Baptist-Mohseni, Victoria Roczyki, Divya Soni, Maurana Brush, Elizabeth Lovell, Stephanie Sanger, M. Constantine Samaan, Russell J. de Souza, Lehana Thabane, Zainab Samaan

**Affiliations:** ^1^Medical Sciences Graduate Program, McMaster University, Hamilton, ON, Canada; ^2^Department of Psychiatry and Behavioural Neurosciences, McMaster University, Hamilton, ON, Canada; ^3^Department of Health Research Methods, Evidence and Impact, McMaster University, Hamilton, ON, Canada; ^4^Undergraduate MD Program, McMaster University, Hamilton, ON, Canada; ^5^Life Sciences Undergraduate Program, McMaster University, Hamilton, ON, Canada; ^6^Department of Biology, McMaster University, Hamilton, ON, Canada; ^7^McMaster Integrative Neuroscience Discovery & Study Program, McMaster University, Hamilton, ON, Canada; ^8^Department of Psychology, Neuroscience and Behaviour, McMaster University, Hamilton, ON, Canada; ^9^Arts & Sciences, McMaster University, Hamilton, ON, Canada; ^10^Health Sciences Undergraduate Program, McMaster University, Hamilton, ON, Canada; ^11^Postgraduate Psychiatry Program, McMaster University, Hamilton, ON, Canada; ^12^Health Science Library, McMaster University, Hamilton, ON, Canada; ^13^Division of Pediatric Endocrinology, McMaster Children’s Hospital, Hamilton, ON, Canada; ^14^Biostatistics Unit, St Joseph’s Healthcare—Hamilton, Hamilton, ON, Canada

**Keywords:** opioids, prescription, opioid use disorder, systematic review, meta-analysis

## Abstract

**Objective:**

Prescription opioid misuse has led to a new cohort of opioid use disorder (OUD) patients who were introduced to opioids through a legitimate prescription. This change has caused a shift in the demographic profile of OUD patients from predominantly young men to middle age and older people. The management of OUD includes medication-assisted treatment (MAT), which produces varying rates of treatment response. In this study, we will examine whether the source of first opioid use has an effect on treatment outcomes in OUD. Using a systematic review of the literature, we will investigate the association between source of first opioid introduction and treatment outcomes defined as continuing illicit opioid use and poly-substance use while in MAT.

**Methods:**

Medline, EMBASE, CINHAL, and PsycInfo were searched from inception to December 31^st^, 2019 inclusive using a comprehensive search strategy. Five pairs of reviewers conducted screening and data extraction independently in duplicate. The review is conducted and reported according to the PRISMA guidelines. A random-effects model was used for meta analyses assuming heterogeneity among the included studies.

**Results:**

The initial search results in 27,345 articles that were screened, and five observational studies were included in the qualitative and quantitative analyses. Our results found that those who were introduced to opioids through a legitimate prescription were significantly less likely to have illicit opioid use (0.70, 95% CI 0.50, 0.99) while on MAT. They were also less likely to use cannabis (0.54, 95% CI 0.32, 0.89), alcohol (0.75, 95% CI 0.59, 0.95), cocaine (0.50, 95% CI 0.29, 0.85), and injection drug use (0.25, 95% CI 0.14, 0.43) than those introduced to opioids through recreational means.

**Conclusion:**

This study shows that the first exposure to opioids, whether through a prescription or recreationally, influences prognosis and treatment outcomes of opioid use disorder. Although the increased pattern of prescribing opioids may have led to increased OUD in a new cohort of patients, these patients are less likely to continue to use illicit drugs and have a different prognostic and clinical profile that requires a tailored approach to treatment.

**Systematic Review Registration:**

PROSPERO CRD42017058143.

## Introduction

North America is experiencing an opioid crisis in which the illicit use of opioids is at an all-time high. Opioids are a class of drugs that are often prescribed to relieve pain and can be highly addictive (1). They include licit substances such as oxycodone, Percocet, hydromorphone, and street drugs such as heroin. The Center for Disease Control (CDC) reports that in the United States, approximately 115 people die every day from an opioid-related overdose (2). In 2017 alone, more than half the drug-related deaths in the States were due to opioids (2). Opioids are controlled substances and are classified by Drug Enforcement Administration (DEA) into various classes according to their abuse potential and medical utility (3). Opioids such as heroin are a Schedule 1 substance indicating high abuse potential and no medical utility, and fentanyl, oxycodone being Schedule II (3). In response to the opioid crisis, Substance Abuse and Mental Health Services Administration (SAMHSA) conducted a national survey and revealed that over 2.1 million people are suffering from an opioid use disorder (OUD) involving prescriptions opioids alone (4). OUD, previously classified as opioid abuse and dependence, is a disorder that affects the psychological, social and physical aspects of an individual’s life (5). Dependence to a substance (i.e. opioids) typically refers to a physical response in the form of withdrawal symptoms when an individual stops using that substance (6). Addiction refers to not being able to resist the urge to use a substance despite there being negative consequence (6). OUD encompass opioid addiction and dependence that signify a problematic use of opioids impacting health and social functioning (5) Withdrawal symptoms experienced due to OUD may include sweating, shakes, anxiety, irritability, and restlessness amongst others (7).

There are several treatments available for OUD which include pharmacological and psychological options. Medication-Assisted Treatment (MAT) includes opioid agonist, antagonists, and partial agonists (8). Some of the more frequently used MATs for OUD are naltrexone, buprenorphine, and methadone (8). Methadone, a synthetic opioid agonist, is one of the most common MAT for treating OUD (8, 9). While research investigating the effectiveness of methadone maintenance treatment (MMT) has shown that it can reduce opioid cravings as well as other symptoms related to opioid withdrawal (i.e. shakes, sweating) through acting on the opioid receptors (8, 10), there is still a high degree of variability for treatment outcomes between individuals such as treatment retention (11–13). Research has suggested that some of this variability may be related to age (14), sex (15), and gender (16) but outcomes are also likely influenced by the increasing prevalence of prescription opioid misuse (17–19).

Current research is suggesting that one reason for the opioid epidemic is the rise of prescription opioid misuse. In 2016 alone, Canada and the United States prescribed over 440 million opioids to patients (20, 21). The National Institutes on Drugs Abuse (NIDA) suggest that anywhere from 8 to 12 percent of individuals prescribed opioids are at risk of developing OUD (22). With the rise of prescription opioid misuse, this has led to a shift in the profile of the “typical” illicit opioid user. Twenty years ago, this demographic profile would have consisted of primarily males in their 20s, misusing heroin intravenously (23, 24) but now, we are seeing a separate cohort of incoming OUD patients that are female, older in age and misusing prescription opioids (25, 26). Prescription medications including opioids are available on the illegal drug market through diversion (27, 28). Diversion of prescription medications may occur at any level from the direct pharmaceutical manufacturing site to patients selling the prescriptions themselves. This has been occurring for many decades for many types of substances (i.e. opioids, benzodiazepines) and with prescription opioids being readily available on the illegal drug market, this has contributed to a demographic shift.

This change in the demographic is substantial because there is evidence that suggests that different types of opioids users have varying experiences while in MAT (29). Previous research suggests that opioid prescription users differ in their treatment outcomes compared to individuals who used heroin (29). Additionally, there is also support for the idea that poly-substance use differs within the OUD population receiving treatment. Poly-substance use has been suggested as a factor that is associated with decreased abstinence from opioids, treatment retention, and related to methadone-related mortality (30–33). Recent research found that cocaine, alcohol, and other substances were used significantly more by heroin users than prescription users (34). Prescription opioid users attending pharmacological treatment for OUD also had significantly longer treatment retention in comparison to heroin users (35). However the previous research is inconclusive as other studies suggested that there is no significant difference in treatment outcomes between prescription introduced and recreational opioid users (36). The magnitude to which this demographic shift has impacted treatment outcomes in specific MAT patient groups has yet to be investigated in a systematic way, and there are conflicting findings in the current literature.

Additionally, there are new, synthetic opioids (i.e. designer fentanyl and its’ analogs) that are available on the street and have been found to be mixed in other illicit substances such as cocaine, methamphetamines and heroin (37). There has been an 88% increase in synthetic opioid-related deaths from 2013 to 2016 whereas the number deaths due to heroin alone use seem to remain consistent (38–40). Prescription opioids are also readily available on the illegal drug market through methods such as prescription resales and theft of prescriptions/prescription pads (28). In recent years, various governments have come up with legislative changes to control access and prescribing patterns for opioids (41–43). With there being new types of synthetic opioids and prescription opioids readily available on the street, it is important to examine if method of introduction to opioids impacts OUD treatment outcomes.

The purpose of this review is to examine differences in patients with OUD on MAT by those introduced to opioids through prescription versus by recreational means on outcomes of continued opioid use, poly-substance use and treatment retention.

This review will fill this knowledge gap and aims to have an important impact in how treatments are designed and tailored to various subgroups within the OUD population. Tailored treatments to address specific concerns in this population may improve MAT outcomes.

## Objectives

The aim of this systematic review is to examine if opioid use disorder patients introduced to opioids through legitimate prescription differ in methadone maintenance treatment outcomes in comparison to those that were introduced to opioids through recreational means.

Specifically, we wanted to examine if these two cohorts differed in:

1. Continued opioid use while in MAT
2. Poly-substance use while in MAT
3. Treatment retention while in MAT

## Methods

### Protocol and Registration

This systematic review was conducted to investigate OUD treatment outcomes by comparing those introduced to opioids through legitimate prescriptions and those introduced through recreational means. The Preferred Reporting Items for Systematic Review and Meta-Analysis (PRISMA) guidelines were followed (44). The protocol for this systematic review has been peer reviewed, published previously (45), and registered with PROSPERO CRD42017058143.

### Eligibility Criteria

This review investigates the association between method of introduction to opioids and MAT outcomes in different settings (i.e. hospital, outpatient, community based) by examining published observational cross-sectional and cohort studies, as well as randomized control trials (RCTs). Included studies compared legitimate prescription opioid introduction to recreational opioid introduction, which can be defined as the use of opioids obtained through means outside of a prescription (i.e. family member, street, using another’s prescription)

Studies that failed to measure the initial method of introduction to opioids were not included. Studies that did not assess at least one of the primary or secondary outcomes of illicit opioid use, poly-substance use and treatment retention were excluded. There were no restrictions on age, sex, or **language**.

### Information Sources and Search Strategy

A search strategy was developed by a health science librarian (SS) to search for studies in the EMBASE, MEDLINE, PsycINFO, and Cumulative Index to Nursing and Allied Health Literature (CINAHL) databases. These databases were searched from inception until December 31, 2019. Search terms were related to prescription opioids and opioid use disorder together with their medical subject headings (MeSH) in different combinations. We also did a manual search of the references of relevant articles to identify any studies that may have been missed. The search strategy has been published in the protocol (45). We have also included the search strategy in the Appendix. Please see **Appendix Table 1**.

### Study Selection

Previously established selection criteria were used by five pairs of reviewers in order to independently complete the title and abstract screening and subsequent full-text review of the eligible articles. Both stages of screening were carried out in duplicate. Upon the occurrence of a disagreement on the status of an article eligibility, resolution was reached through discussion to consensus between the pair, or with the consultation of a third party. Inter-rater agreements were established using a kappa statistic, where a kappa value of at least 0.75 is indicative of exceptional agreement between reviewers (46). The mean kappa value between pairs was 0.88.

### Data Collection and Data Items

A piloted data extraction form was used by reviewers to retrieve data in duplicate. These forms extracted information relating to the author, year of publication, journal, and country of publication. Details of the study’s methodology and results were also retrieved. More specifically, information on research design used, demographics of the research participants, type and method of measuring initial type of opioid introduction (i.e. medical prescription or recreational), MMT outcome measures, overall findings of the study, and the study’s statistical results was included. If data pertaining to the aforementioned items was missing, the authors were contacted.

### Risk of Bias of Individual Studies

The risk of bias was independently assessed by two reviewers who reviewed the methodological quality of the eligible studies using the Newcastle-Ottawa Scale (NOS), used mainly for observational studies to assess choice bias, performance bias, identification bias, and information bias (47). A modified model was used that has eliminated items concerning the comparability of groups (48). It consists of 7 questions and is quantified on a scale of 0 to 3, where 0 is high risk of bias and 3 is low risk of bias. The Grading of Recommendations Assessment, Development and Evaluation (GRADE) criteria was utilized to assess the quality and strength of the evidence (49). This is provided in **Table 1**.

**Table 1:** Summary of findings.

		**Illicit opioid use**	**Marijuana use**	**Cocaine use**	**Any injection drug use**	**Alcohol use**	**Benzodiazepine use**
**Certainty assessment**	№ of studies	3	3	3	2	2	2
	Study design	observational studies	observational studies	observational studies	observational studies	observational studies	observational studies
	Risk of bias	not serious	not serious	not serious	not serious	not serious	not serious
	Inconsistency	not serious	not serious	not serious	not serious	not serious	not serious
	Indirectness	not serious	not serious	not serious	not serious	not serious	not serious
	Imprecision	serious^a^	serious^a^	serious^a^	serious^a^	serious^a^	serious^a^
	Other considerations	strong association	strong association	strong association	very strong association	none	none
**№ of patients**	Prescription opioid	339/691 (49.1%)	399/651 (61.3%)	175/651 (26.9%)	122/167 (73.1%)	259/607 (42.7%)	73/551 (13.2%)
	Illicit opioid introduction	309/709 (43.6%)	258/540 (47.8%)	91/540 (16.9%)	32/81 (39.5%)	185/509 (36.3%)	53/500 (10.6%)
**Effect**	Relative (95% CI)	OR 1.42 (1.01 to 2.00)	OR 1.87 (1.12 to 3.12)	OR 2.01 (1.17 to 3.46)	OR 4.07 (2.31 to 7.15)	OR 1.34 (1.05 to 1.71)	OR 1.21 (0.79 to 1.86)
	Absolute (95% CI)	**87 more per 1,000** (from 2 more to 171 more)	**153 more per 1,000** (from 28 more to 263 more)	**121 more per 1,000** (from 23 more to 244 more)	**332 more per 1,000** (from 206 more to 429 more)	**70 more per 1,000** (from 11 more to 131 more)	**19 more per 1,000** (from 20 fewer to 75 more)
**Certainty**		⨁⨁◯◯ LOW	⨁⨁◯◯ LOW	⨁⨁◯◯ LOW	⨁⨁⨁◯ MODERATE	⨁◯◯◯ VERY LOW	⨁◯◯◯ VERY LOW
**Importance**		CRITICAL	IMPORTANT	IMPORTANT	IMPORTANT	IMPORTANT	IMPORTANT

CI, Confidence interval; OR, Odds ratio.

^a^Imprecise as adjusted pooled estimates were not possible to conduct.

### Statistical Analyses

All included studies were qualitatively summarized. A meta-analysis was conducted on the primary outcome of illicit opioid use and the outcome of poly-substance use. Review Manager 5.2 was used to conduct the meta-analyses. The substances included in this were cannabis, alcohol, injection drug use, cocaine, and benzodiazepines. These were the substances that were examined in the included studies. Two of the included studies investigated treatment retention but were unable to be meta-analyzed as they were reported in different ways. The outcomes are presented in a forest plot. The meta-analyses reflect the associations found between the outcomes and method of introduction to opioids (legitimate prescription and recreational). Due to the limited number of studies, we were not able to conduct any subgroup analyses for age, sex, country, and type of MAT treatment.

We have shown our pooled dichotomized data as odds ratio (OR) with 95% confidence intervals. The I^2^ statistic was used to compute heterogeneity. Cochrane suggests that a value of <40% might not signify a noteworthy heterogeneity (50). A random effect model, which considers both within study and between study variance in comparison to the fixed-effect model, was used to account for expected heterogeneity in the included studies. We were not able to conduct an adjusted analysis as covariates were not controlled for. We were unable to examine publication bias as we have less than 10 included papers. Previous studies have reported that it is not possible to assess publication bias with less than 10 studies (51). PRISMA reporting guidelines were followed throughout this process (44).

### Types of Interventions

#### Experimental

The experimental intervention includes those participants that were introduced to opioids through recreational use and are now in MAT for OUD.

#### Comparator

The accepted comparators include those that were introduced to opioids through a legitimate physician’s prescription and are now in MAT for OUD.

### Outcome Measures

#### Continued Opioid Use

We have defined continued opioid use to be the use of any opioids while the patient is in methadone maintenance treatment.

#### Poly-Substance Use

We defined poly-substance use as the use of any of the previously defined substances before or during MMT.

#### Treatment Retention

We defined treatment retention as the length of time a patient stayed in their MAT without dropping out.

## Results

### Study Selection

**Figure 1:**
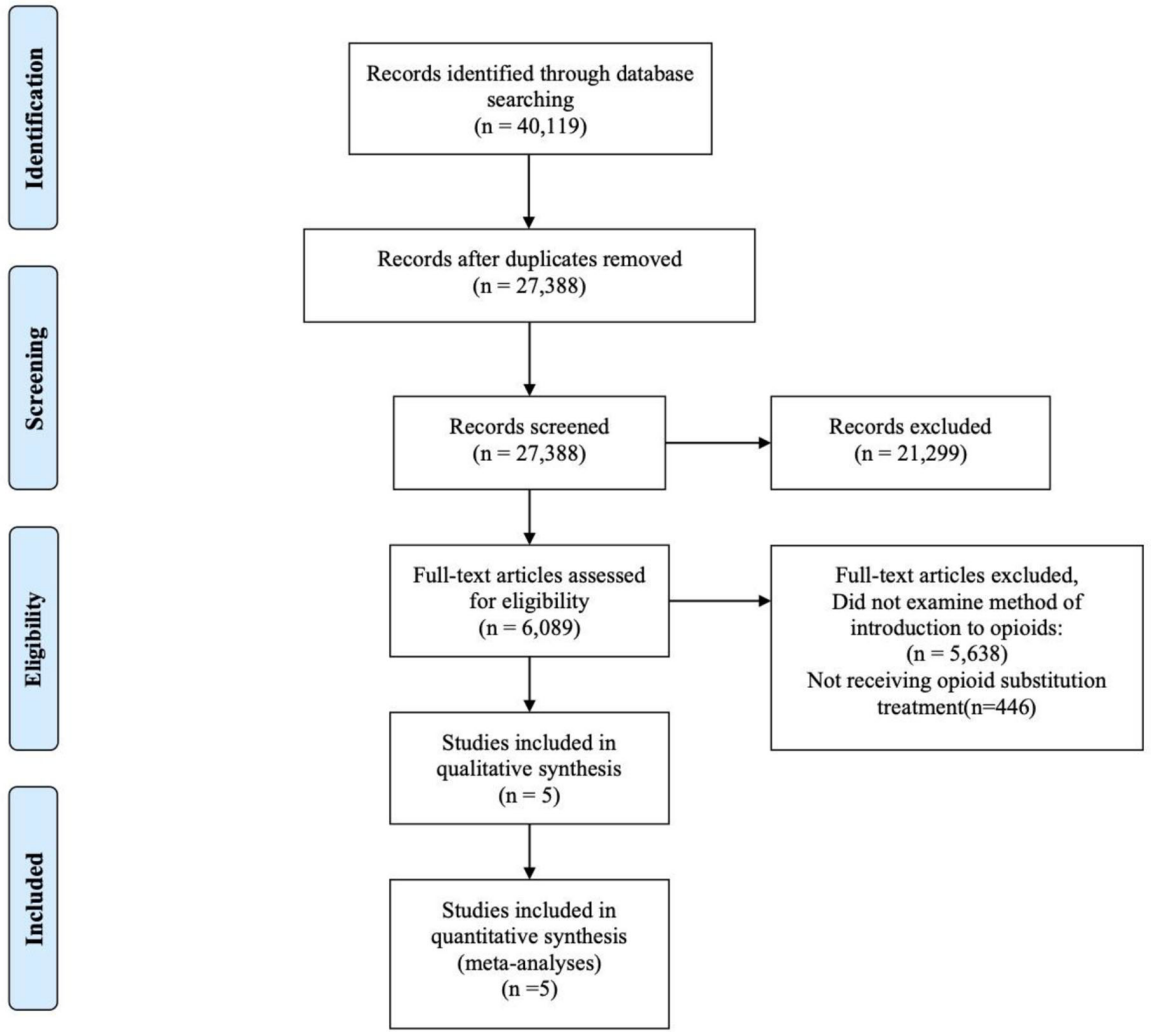
PRISMA Flow Diagram.

From the databases searched, a total of 27,345 articles went through the title and abstract screening process. After removing 3,264 duplicates and 24,076 studies that did not meet the inclusion criteria, a total of five studies were included. **Figure 1** is the PRISMA flow diagram of the screening process. All five studies were included in the meta-analyses of the outcomes. Three out of five studies were subjected to the meta-analysis of the primary outcome of illicit opioid use (36, 52, 53).

### Study Characteristics

The characteristics of the included studies are summarized in **Table 2**. Five papers were included in this systematic review, all of which were observational studies looking at patients in MAT for opioid use disorder. Two studies looked at patients receiving buprenorphine or methadone treatment (36, 54). One study included patients undergoing methadone treatment (53). One study only looked at buprenorphine treated patients (55) while the final study looked at buprenorphine-naloxone patients (52). All five of these studies compared individuals initially introduced to opioids for prescription use with individuals introduced to opioids *via* recreational use. The majority of the sample consisted of male participants (57.4%).

**Table 2:** Summary of characteristics.

Study	Country	Study Design and type of opioid substitution treatment	Participants (sample size in each group, age range, sex, inclusion/exclusion criteria, primary diagnosis)	Physicians prescription and recreational use Definitions	Outcomes (definition and how they were measured)	Statistical Analysis	Results
Canfield et al. (54)	United States	Cross-sectionalType of OST: N/A (patients recruited from inpatient detoxification unit)	N = 75 (physician prescription: n = 31, Illicit opioid: n = 44)Mean age (range): 31.5 (18–70)Sex: 49 male (65%), 26 female (35%)Inclusion criteria: met DSM-IV criteria for opiate dependence, wished to become abstinent from opioids, at least 18 years old, able to understand spoken English, able to provide informed consent, had urine toxicology positive for opiates on day of admissionExclusion criteria: none (other than patient refusal)	Physician prescription: participants who reported that their addiction began with opioids that were prescribed for them (i.e., licit use)Recreational use: participants who traced the onset of their addiction to either diverted prescription medications or from “street drugs” (i.e., illicit drug use)	Collected self-report data related marijuana, cocaine and benzodiazepine use	Fisher exact test for between group comparisons of categorical variables; Student t-test for between group comparisons of continuous variables	First time Licit users were less likely to have ever used marijuana [27/31 (87%) vs. 44/44 (100%) p = 0.026]No significant association found between method of introduction to opioids and use of cocaine or benzodiazepines.
Cooper et al. (36)	Australia	Prospective cohortType of OST: not reported	N = 108 (physician prescription: n = 41, illicit opioid: n = 67)Mean age: 41 (range not reported)Sex: 52 male (48%), 56 female (52%)Inclusion criteria: had entered treatment for pharmaceutical opioid dependence, were competent in EnglishExclusion criteria: not reported	Participants were classified as having “iatrogenic dependence” if their first opioids of concern were prescribed by a doctor for a legitimate medical reason	Collected self-report data on participants’ opioid use history (including past month illicit opioid use)Injection drug use history (including heroin, non-medicinal/non-prescribed opioids)Treatment retention was reported as median number of years on treatment	χ^2^ tests, independent *t-*tests, and Mann-Whitney *U* tests used to examine baseline differences between those who initiated opioid use for iatrogenic and non-iatrogenic reasons	No significant difference between iatrogenic dependence vs. non-iatrogenic dependence in unsanctioned opioid use in the past month [19.5 vs. 25.4%, odds ratio 0.71, 95% CI (0.28, 1.84)]Iatrogenic dependence associated with a lower prevalence of lifetime injection of any drug [41.5 vs. 68.7%, odds ratio 0.32, 95% CI (0.14, 0.73)]No significant difference between iatrogenic dependence vs. non-iatrogenic dependence in median length on current treatment, p = 0.739
Dreifuss et al. (52)	United States	Cross-sectionalType of OST: sublingual buprenorphine/naloxone	N = 360 (physician prescription: n = 199, illicit opioid: n = 117)Mean age: 32.5 (range not reported)Sex: 209 male (58%), 151 female (42%)Inclusion criteria: met DSM-IV criteria for current opioid dependence; were at least 18 years old; unsuccessful in Phase 1 of POATS study (returned to opioid use) and subsequently enrolled in Phase 2Exclusion criteria: heroin use on ≥4 days in past month; lifetime diagnosis of opioid dependence due to heroin alone; history of ever injecting heroin; concurrent formal ongoing substance abuse treatment	Physician prescription: first obtained opioids *via* a legitimate prescriptionRecreational use: given their first opioids by someone, or initially bought them from a drug dealer	Substance Use Report (corroborated by weekly urine drug screens) administered weekly duringtreatment and every two weeks during follow-up as primary measure todetermine “successful outcome” in Phase 2 (abstinence from opioids during final week of treatment and ≥2 of 3 weeks prior)	Bivariate analyses compared patients who were successful at end of treatment with those who were not	Patients who first used opioids to relieve physical pain were more likely to succeed (have a successful outcome of abstinence from opioids), while those who had first used to get high were less likely to do so
Sanger et al. (53)	Canada	Prospective CohortType of OST: methadone maintenance treatment	N = 976 (physician prescription: n = 469, illicit opioid: n = 507)Mean age: 40.8 in physician prescription group, 36.9 in illicit opioid group (ranges not reported)Sex: 535 male (54.8%), 441 female (45.2%)Inclusion criteria: over 18 years of age; met DSM-IV criteria for opioid dependence (modified in DSM-5 to opioid use disorder); on methadone maintenance treatment; able to provide informed, written consent, undergo urine drug screens, and provide information on source of initiation to opioidsExclusion criteria: receiving an alternate opioid substitution therapy; currently taking prescription opioids; currently on suboxone; unable to provide a urine sample	Physician prescription: initial exposure to opioids through a medical prescriptionRecreational use: initial exposure to opioids through other means including at home, family member, street, school, or friend	Maudsley Addiction Profile (MAP) administered to measure specific details of self-reported drug use for cocaine, cannabis, alcohol, and benzodiazepine,Illicit opioid use measured by regular urine drug screens at baseline and 6-month follow-upTreatment retention was reported as mean number of months on treatment	Multivariable logistic regression used to examine relationship between illicit drug use and treatment retention in relation to source of initial opioid use	Those initiated *via* prescription were less likely to have used cannabis (OR = 0.66, 95% CI 0.49–0.90, P = .008) in comparison to those introduced by recreational meansNo significant association between method of introduction and illicit opioid use, cocaine, alcohol, benzodiazepine use.No significant association between method of introduction and current length of treatment
Tsui et al. (55)	United States	Cross-sectionalType of OST: buprenorphine	N = 140 (physician prescription: n = 40, illicit opioid: n = 100)Mean age: 38 (range not reported)Sex: 106 male (76%), 34 female (24%)Inclusion criteria: age 18–65; DSM-IV diagnosis of opioid dependence; Hamilton Depression Revised Scale (MHDRS) score > 14; absence of significant suicidal ideation; willingness and ability to complete 3-month treatment with buprenorphine; no history of severe mental illness (bipolar disorder, schizophrenia, schizoaffective, or paranoid disorder); no currently prescribed medications for depression (participants not specifically excluded if taking tricyclic anti-depressant only for pain); ability to complete the study assessment in EnglishExclusion criteria: NR	Participants’ responses to the question: “Who introduced you to opiates?” (possible responses included physician, sexual partner, friend, family member, stranger, and no one)	Collected self-report data on current (last 30 days) and past use of prescription opioids and heroin (including route of administration) using Addiction Severity Index (ASI)Collected self-report data on regular use of alcohol, marijuana and cocaine by asking, “Prior to starting opiates, did you ever have daily or regular use of (drug)?”	Descriptive analyses comparing individuals who reported physician introduction to opioids to those who did not report physician introduction; examined differences in demographic, clinical, and substance use-related variables between participants using Student t-tests and Pearson chi-square tests	Participants introduced by physician were more likely to be currently using prescription opioids only, less likely to have injected drugs (38 vs. 76%, p < 0.01), half as likely to currently inject drugs (28 vs. 57%, p < 0.01), and significantly less likely to report prior use of marijuana (53 vs. 72%, p = 0.03) and cocaine (23 vs. 45%, p = 0.01)Regular use of alcohol prior to starting opioids was equally reported among those who were and were not introduced by a physician to opioids

Three out of five studies looked at the primary outcome of illicit opioid use (36, 52, 53). Two studies examined injection drug use (36, 55), three studies examined cannabis use, two studies examined alcohol use (53, 55), two studies examined benzodiazepine use (53, 54), and three studies examined cocaine use (53–55). Additionally, two studies examined treatment retention (30, 32).

### Risk of Bias Within Studies

**Figure 2:**
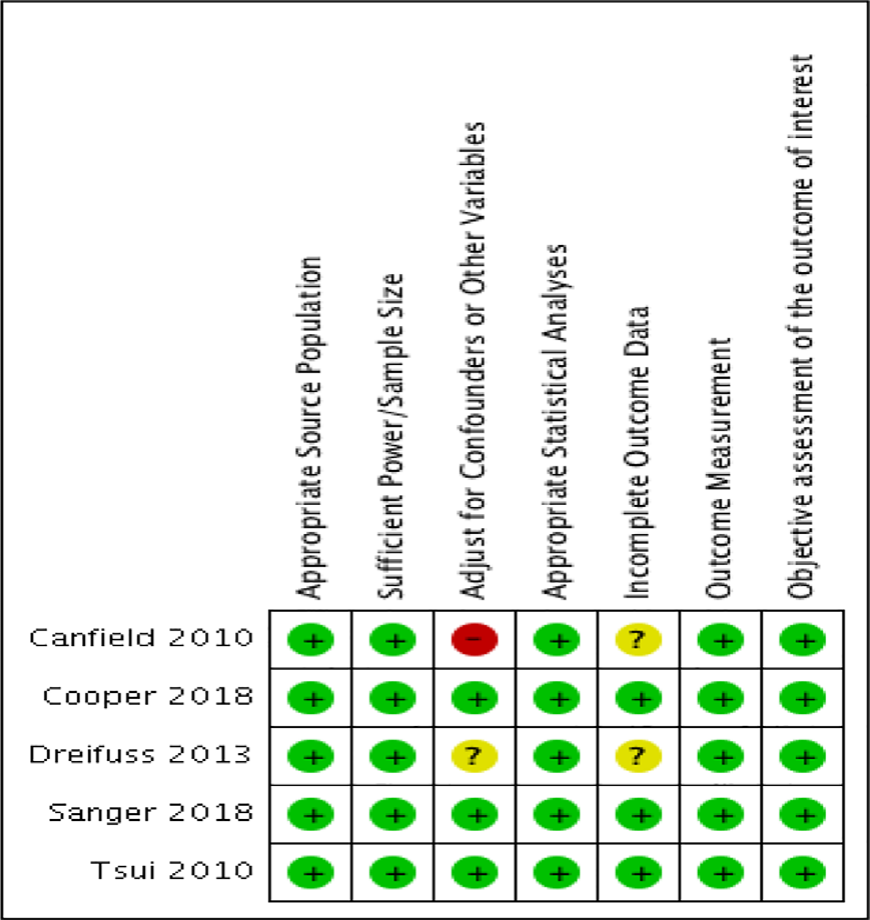
Risk of bias summary: review authors' judgements about each risk of bias item for each included study.

The quality of the studies included are shown in **Figure 2**. Justifications for assessments are presented in **Appendix Table 1** with the risk of bias tables. The modified NOS was used to rate the internal validity of the studies shown in **Figure 2**, and assess the quality of these observational studies (47, 48). Generally, most of the studies included have relatively low to moderate risk of bias, except for one (54). Specifically, this study shows a high risk of bias when adjusting for confounders or other variables as the researchers did not adjust for confounders, instead opting to perform student t-tests. Another study also shows an unclear risk of bias when adjusting for confounders or other variables since the information they provide is unclear (52). Two of the studies included show an unclear risk of bias in terms of incomplete outcome data, simply because they do not provide any information about this (52, 54). Aside from these biases, all five of the observational studies were generally well reported on all other characteristics, including appropriate source population, sufficient power and sample size, appropriate statistical analysis, valid outcome measurement, and objective assessment of the outcome of interest.

### Results of Individual Studies

#### Illicit Opioid Use

Our meta-analysis pooled results from three studies comparing the continuation of opioid use among individuals first introduced to opioids by a legitimate prescription vs. a recreational source. Cooper et al. (36) collected self-reported data on past month and lifetime opioid use. We used the data provided on the past month opioid use. Dreifuss et al. (52) collected data on the continued use of opioids using weekly substance use reports and urine drug screens. Sanger et al. (53) used urine drug screens to investigate illicit opioid use. The remaining two studies did not report on the outcome of continued opioid use (54, 55). Canfield et al. (54) examined progression of opioid use over time, but not as an outcome of the means of opioid use introduction. Tsui et al. (55) reported on the different patterns in type of opioids the groups would use (i.e. prescription, street drugs, or both) but did not provide information pertaining to the exact number of patients that were currently using opioids between licit and illicit method of introduction groups.

**Figure 3:**
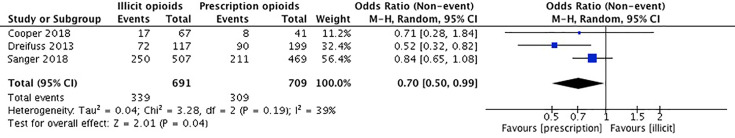
Forest Plot for Illicit Opioid Use.

The studies included in our meta-analysis comprise a total sample size of 1,400 participants. Cooper et al. (36) reported that those introduced through a prescription were associated with a lower prevalence of lifetime heroin use, but no difference in past-month illicit opioid use. Dreifuss et al. (52) found that those introduced to opioids by means of a prescription were associated with discontinued opioid use in the final weeks of treatment, whereas those introduced through illicit means were associated with continued opioid use in treatment. In Sanger et al. (54), there was no significant association between the source of opioid introduction and continued opioid use. We conducted an unadjusted analysis using odds ratios to compared continued opioid use during treatment among those who were first introduced to opioids through a prescription versus an illicit source. We found that individuals who were introduced to opioids through prescription means were significantly 70% less likely to have continued to use opioids while in MAT (OR 0.70, 95% CI 0.50, 0.99, p-value 0.04). Please see **Figure 3**.

#### Injection Drug Use

Our meta-analysis pooled results from two studies comparing injection drug use among participants first introduced to opioids through a prescription versus an illicit source. Cooper et al. (36) collected self-reported data on injection drug use history. Tsui et al. (55) used the Addiction Severity Index (ASI) to collect self-reported data on current and past use of prescription opioids and heroin, including the route(s) of administration. The remaining three studies did not report on the outcome of injection drug use (52–54). Canfield et al. (54) reported a combination of intranasal and intravenous routes of administration and intravenous drug use could not be extrapolated. Dreifuss et al. (52) and Sanger et al. (53) did not report any data on intravenous drug use.

**Figure 4:**
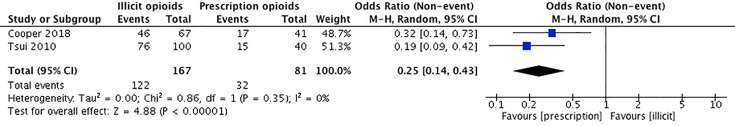
Forest Plot for Any Injection Drug Use.

The studies included in our meta-analysis comprise a total sample size of 248 participants. In Cooper et al. (36), those introduced to opioids through a prescription have a lower prevalence of any injection drug use. Tsui et al. (55) reported that those introduced to opioids by a physician were less likely to have any injection drug use. We conducted an unadjusted analysis using odds ratios to compare any injection drug use among those who were introduced to opioids through a prescription vs. an illicit source. We found that individuals who were introduced to opioids through a prescription source were significantly less likely to engage in injection drug use in comparison to those introduced through recreational means (OR 0.25, 95% CI 0.14, 0.43, p-value < 0.001). Please see **Figure 4**.

#### Cannabis Use

Our meta-analysis pooled results from three studies comparing cannabis use in the initiation source of opioid use, by means of prescription vs. an illicit source. Canfield et al. (54) collected self-reported data on cannabis use history. Sanger et al. (53) used the Maudsley Addiction Profile (MAP) to acquire self-reported data on cannabis use in the past 30 days. Tsui et al. (55) acquired self-reported data on regular use of cannabis. The remaining two studies did not report on the outcome of cannabis use (36, 52).

**Figure 5:**
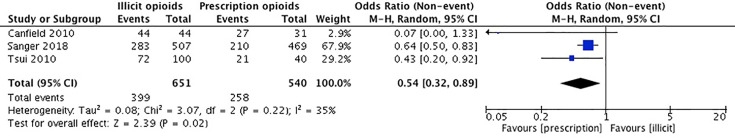
Forest Plot for Cannabis Use.

The studies included in our meta-analysis comprise a total sample size of 1,191 participants. In Canfield et al. (54), participants who were first introduced to opioids by means of a prescription were less likely to have ever used cannabis. Sanger et al. (53) reported that those first introduced to opioids by a prescription were less likely to have used cannabis in the past 30 days than those first introduced to opioids by a recreational source. In Tsui et al. (55), participants who were introduced to opioids by a physician were less likely to report prior use of cannabis. We conducted an unadjusted analysis using odds ratios to compare cannabis use among those who were introduced to opioids by a prescription versus an illicit source. We found that those who initiated the use of opioid(s) through a prescription source were significantly less likely to use cannabis (OR 0.54, 95% CI 0.32, 0.89, p-value 0.02). Please see **Figure 5**.

#### Alcohol Use

Our meta-analysis pooled results of two studies comparing the effect of opioid introduction on alcohol use. Sanger et al. (53) used the MAP to acquire self-report data on alcohol use within the past 30 days. Tsui et al. (55) collected self-report data on regular use of alcohol by asking participants the question “prior to starting opiates, did you ever have daily or regular use of alcohol?”. The remaining three studies did not report on the outcome of alcohol use (36, 52, 54). Cooper et al. (36) asked participants about injection use of alcohol and reported their results as a measure of injection history of any drug. Dreifuss et al. (52) examined alcohol use as a predictor of treatment success but not as an outcome of initial exposure to opioids. Canfield et al. (54) did not report any data on alcohol use.

**Figure 6:**
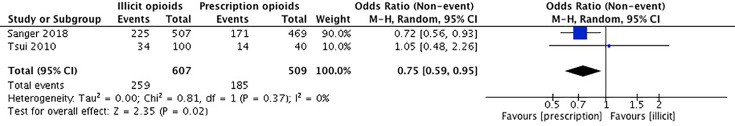
Forest Plot for Alcohol Use.

The studies included in our meta-analysis comprise a total sample size of 1,116 participants. In Sanger et al. (53), there was no significant association between source of opioid initiation and alcohol use. In Tsui et al. (55), there was no significant difference in regular use of alcohol prior to opioids between those who were introduced to opioids by a physician versus those who were not. For this meta-analysis, we used the results for the entire population from both Sanger et al. (53) and Tsui et al. (55). We conducted an unadjusted analysis using odds ratios to compare alcohol use among those who first initiated opioids through a prescription versus an illicit source. We found that individuals who were introduced to opioids through a legitimate prescription were significantly less likely to have used alcohol (0.75, 95% CI 0.59, 0.95) (OR 0.75, 95% CI 0.59, 0.95, p-value 0.02). Please see **Figure 6**.

#### Cocaine Use

Our meta-analysis pooled results of three studies investigating cocaine use. Canfield et al. (54) collected self-reported data on any previous cocaine use. Sanger et al. (53) used the MAP to acquire self-report data on cocaine use within the past 30 days. Tsui et al. (55) collected self-report data on regular use of cocaine by asking participants the question “prior to starting opiates, did you ever have daily or regular use of cocaine?”. The remaining two studies did not report on the outcome of cocaine use (36, 52). Cooper et al. (36) collected data on the use of cocaine only in the context of injection drug use and reported their results as a measure of injection history of any drug. Dreifuss et al. (52) examined cocaine use as a predictor of treatment success but not as an outcome of initial exposure to opioids.

**Figure 7:**
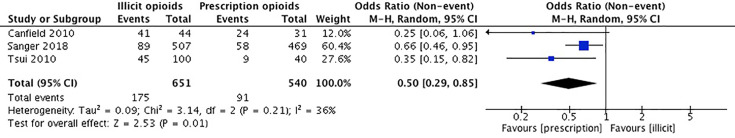
Forest Plot for Cocaine Use.

The studies included in our meta-analysis comprise a total sample size of 1,191 participants. In Canfield et al. (54), there was no significant difference in use of cocaine between those who reported obtaining their first opioid through a prescription versus an illicit source. In Sanger et al. (53), there was no significant association between source of opioid initiation and cocaine use. In Tsui et al. (55), participants who were first introduced to opioids by an illicit source were significantly more likely to report prior use of cocaine. For this meta-analysis we conducted an unadjusted analysis using odds ratios to compare cocaine use among those who first initiated opioids through a prescription versus an illicit source. We found that individuals who were introduced to opioids through prescription were significantly less likely to use cocaine (OR 0.50, 95% CI 0.29, 0.85, p-value 0.01). Please see **Figure 7**.

#### Benzodiazepine Use

Our meta-analysis pooled results of two studies comparing benzodiazepine use among participants first introduced to opioids through a prescription versus an illicit source. Canfield et al. (54) collected self-report data on any previous benzodiazepine use. Sanger et al. (53) used the MAP to acquire self-report data on benzodiazepine use in the past 30 days. The remaining three studies did not report on the outcome of benzodiazepine use (36, 52, 55). Cooper et al. (36) collected data on previous injection use of benzodiazepines and reported their results as a measure of injection history of any drug. Dreifuss et al. (52) examined the use of sedatives as a predictor of treatment success but did not specifically assess benzodiazepine use as an outcome of initial exposure to opioids. Tsui et al. (55) did not collect any data on benzodiazepine use.

**Figure 8:**
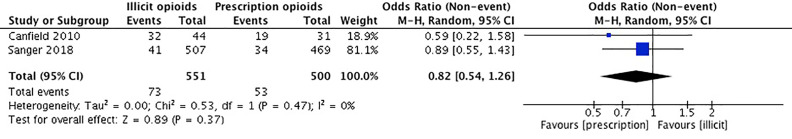
Forest Plot for Benzodiazepine Use.

The studies included in our meta-analysis comprise a total sample size of 1,051 participants. In Canfield et al. (54), there was no significant difference in benzodiazepine use among those who reported obtaining their first opioid through a prescription vs. an illicit source. In Sanger et al. (53), there was no significant association between source of opioid initiation and benzodiazepine use. We conducted an unadjusted meta-analysis using odds ratios to compare benzodiazepine use among those who first initiated opioids through a prescription vs. a recreational source. We found that there was no significant association between individuals who were introduced to opioids through prescription and those that were introduced through recreational means (OR 0.82, 95% CI 0.54, 1.26, p-value 0.37). Please see **Figure 8**.

#### Treatment Retention

Two studies examined treatment retention (36, 53) however we were unable to combine study results to conduct a meta-analysis. Sanger et al. (53) examined the mean length in treatment and found that there was no significant association between the prescription introduction and recreational introduction groups (53). Cooper et al. (36) reported the length of treatment in median years. They reported no significant association between those introduced to opioids through a prescription in comparison to those introduced by recreational means for length of current treatment in median years (36).

### Risk of Bias Across Studies

**Figure 9:**
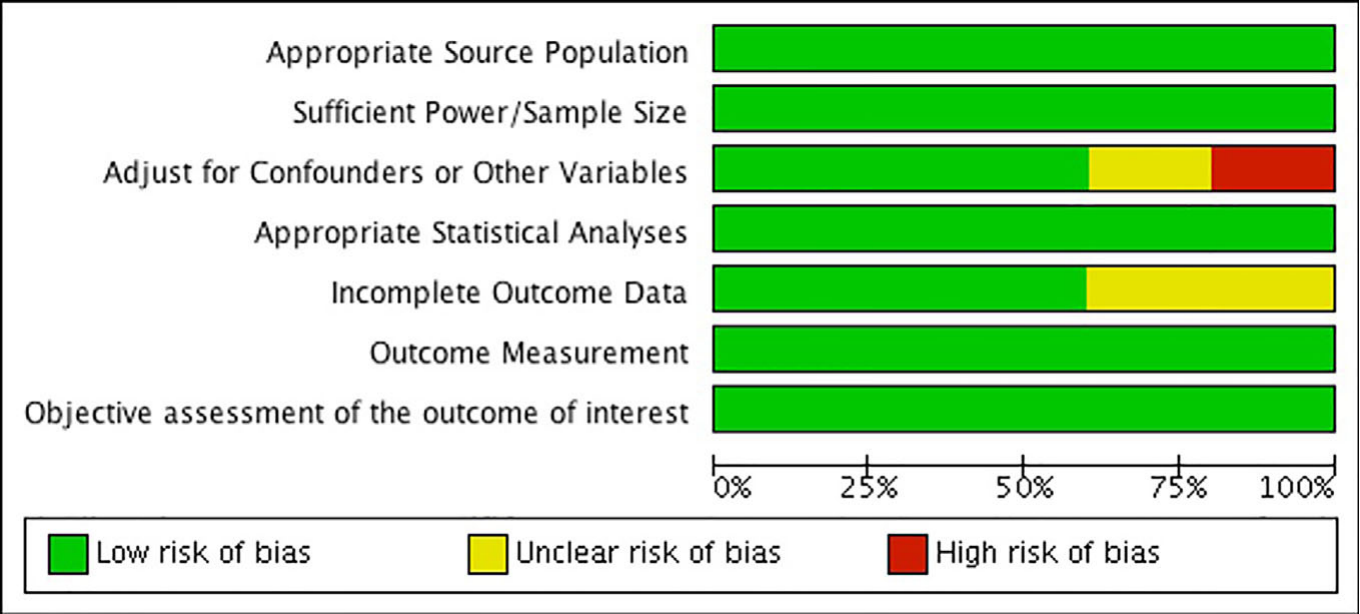
Risk of bias graph: review authors' judgements about each risk of bias item presented as percentages across all included studies.

When assessing risk of bias across studies (**Figure 9**), we noticed a few trends. First, two of the studies show an unclear or high risk of detection bias, which indicates that the studies either did not adjust for confounders and other variables, or did not properly report that they did so (52, 54). Secondly, two of the studies also show an unclear risk of detection bias as they fail to provide outcome data, or the data provided is unclear (52, 54). Overall, our findings show that the results from these two observational studies should be interpreted carefully due to risk of bias. Further, our results show that the other three observational studies were generally well reported and bias free (36, 53, 55). Please see **Figure 9**.

### Additional Analysis

As there were a small number of studies included in this review, it was not possible to conduct any additional analyses.

## Discussion

### Summary of Evidence

Opioid use disorder is a serious illness that affects approximately 26 to 36 million people across the globe (2). Not only does this illness affect the individual in multiple aspects of their lives, it places a great economic burden on healthcare systems (56). We have recently seen a dramatic increase in the number of people misusing opioids, a significant proportion of whom misuse prescription opioids specifically. While this crisis has global impacts, North America has experienced the majority of the burden of illness. The United States alone consumes 80% of the global supply of prescription opioids, and it is estimated that their use has increased by 300% since 1991 (57). Research has suggested that those prescribed an opioid prescription for chronic pain have a risk of up to 60% of misusing prescriptions (58). It is critically important to investigate the emerging cohort of patients who were introduced to opioids by legitimate prescriptions to see whether they fare differently in MAT compared to those who were introduced to opioids recreationally. To our knowledge, this is the first systematic review to synthesize the literature examining this question.

Our meta-analysis found that those that were introduced to opioids through a legitimate prescription were less likely to use illicit opioids while in treatment than those that were introduced to opioids through recreational means (OR 0.70, 95% CI 0.50, 0.99, p-value 0.04). Our findings also revealed that the prescription introduction to opioids cohort were less likely to have used cocaine (OR 0.50, 95% CI 0.29, 0.85, p-value 0.01), alcohol (OR 0.75, 95% CI 0.59, 0.95, p-value 0.02), cannabis (OR 0.54, 95% CI 0.32, 0.89, p-value 0.02), and injection drugs (OR 0.25, 95% CI 0.14, 0.43, p-value <0.001). There was no association found between the source of introduction to opioids and benzodiazepine use (OR 0.82, 95% CI 0.54, 1.26, p-value 0.37).

Those introduced to opioids through prescriptions were found to be less likely to continue using opioids during treatment than those whose first introduction was through recreation. This suggests that first introduction to opioids through illegal means predicts continued use during treatment, and that the first introduction may explain trends in subsequent opioid use. Brands et al. demonstrated that patients in MMT who used only prescription opioids had significantly less experience with sharing opioid injection equipment in comparison to those patients who used heroin only or initially (59). While this study did not ask patients about their first introduction to prescription opioids, most patients using prescription opioids only (86%) or initially (61.9%) indicated that their initial reason for using opioids was to manage pain. They conclude that those who were likely introduced to opioids through prescription as a means of treating pain tend to engage in less risk-taking behavior, and are less likely to continue using opioids during treatment in comparison to those not using opioid drugs to manage pain (59). Further, in another study of patients in treatment for OUD, those using only prescription opioids had a higher treatment retention, fewer opioid-positive urine samples, and were more likely to complete treatment than those patients using a combination of heroin and prescription opioids or those using heroin exclusively (35). Taken together, first introduction and reason for use, perhaps mediated by risk-taking behaviors, may predict future opioid use and explain our finding that those who were not first introduced to opioids through a prescription have an increased likelihood of continued use in treatment. People whose opioid use was first initiated through prescription also tend to be demonstrate lower risk-taking behavior, further supporting the observation that those who initiate opioid use from a prescription tend to be less likely to continue use during treatment. Prescription-introduced opioid users are more likely to be female, generally have an older age of opioid use onset, and are more like to have completed a post-secondary education (53). These factors likely influence the level of continued use of illicit opioids in treatment as women in general are less likely to use opioids (60) and are shown to engage in fewer risks than men in terms of both everyday risk-taking behaviors (61) as well as in financial, recreational, ethical, and recreational domains (62). Risk-taking attitudes are found to be reduced with age (62), and older adults are also less likely to partake in risk-taking behavior and illegal opioid use while in treatment. A study of treatment outcomes for opioid use found that 61% of older adults had no positive urine screens for opioids, compared to 35% in younger adults after initiating treatment (63).

Our finding that those introduced to opioids through recreational means are more likely to engage in using other substances such as alcohol, marijuana, and cocaine, is also congruent with the literature. Studies have found that the nonmedical use of opioids was significantly associated with the use of other illicit substances (56). Specifically, there is research that suggests that there are differences in polysubstance use between prescription users and recreational users, and that this poly-substance use in recreational opioid users may be associated with risk-taking behaviors. A study investigating HIV risk-taking behavior found that men who are recreational, poly-substance drug users were more likely to engage in risky behaviors such as the sharing of needles and sex without protection (64). Morely et al. took a closer look at recreational drug users and found that different mental disorders and behavior patterns are predictive of the type and degree of polysubstance use a recreational user engages in (65). Depression and anxiety disorders were found to be predictive of medication and cannabis use, whereas violent and risky behavior suggested the use of illicit or all drugs. In contrast, participants in the non-polysubstance class were more likely to be female, have a lower desire to use drugs, and were less likely to have a diagnosis of anxiety or depression, or engage in violent risk-taking behaviors. Thus, risk-taking behavior and the presence of mental illness may be predictive of polysubstance use in recreational drug users, which would explain our finding that recreational drug users have a higher likelihood of misusing more than one illicit substance. A study reported that respondents who had experienced at least one major depressive episode in the past year were more likely to engage in non-medical use of prescription pain relievers (66). Providing support and resources for comorbid mental health concerns within this population may be an area that clinicians and policy makers should consider implementing within OUD treatment plans.

With the increased availability of prescription opioids contributing to the opioid epidemic, countries across the globe have taken initiatives to control access and prescribing patters of opioids. Some of these initiatives include legislative changes through guideline recommendations in opioid prescribing for chronic, non-cancer pain, acute pain conditions, and prescription monitoring programs (42, 67). Research examining these changes have suggested that there is a decrease in opioid prescribing with these measures in place such as using the recommendation of nonsteroidal anti-inflammatory drugs (NSAIDs) over opioids for acute pain (28, 67–70). These findings in combination with the ever-changing synthetic opioids drug market would suggest that is important to continue to tailor recommendations to fit the ever-changing opioid user.

These findings are important as they can help develop tailored MAT programs for patients. It may be important to consider comorbid medical conditions such as pain that may have led to being introduced to opioid by prescription or concurrent substance use when creating a treatment plan. This systematic review has highlighted that those introduced to opioids by prescription means are less likely to use other substances including opioids. This cohort of individual are most likely people that did not intend to engage in risk-taking behavior. They ended up dependent to opioids because of the associated addictive properties. They may benefit from being treated in different settings and with the use of different approaches to addiction philosophy. Addiction specialists should consider addressing harm reduction strategies such as hepatitis C treatment awareness and provision of clean needles to those still engaging is IV drug use while in treatment. Pain specialists and pharmacists may want to consider including a brief educational component and treatment plan to mitigate problematic use potential surrounding opioids when prescribing opioids to a patient is necessary. Additionally, those who were introduced through recreational means likely have a different set of problems to address than those whose use began with prescriptions. Perhaps there should be additional support provided for patients that desire to stop using additional substances alongside illicit opioids. The current lack of data present on poly-drug use, the associated risks and individual goals is limited and should be expanded on in order to develop personalized support for poly-drug users. Some research has predicted that the increased strictness of prescribing opioids will not have a huge impact on the number of opioid overdoses and deaths (71). Targeting illicit opioid use in treatment is where focus should also be. Policy makers may want to provide different treatment settings for OUD patients and, by identifying patients with high risk behavior patterns who were introduced to opioids recreationally, can take advantage of opportunities for interventions to reduce patients’ hazardous use of other substances. It is also important to address the lack of information on the emergence of novel opioid substances and their apparent popularity with illicit opioid users as it limits the level of insight current literature can provide to drug addiction services and clinicians. Due to the lack of information on current opioid related changes future directions may include updating this paper to possibly highlight novel data on poly-drug use and opioid derivatives. Furthermore, due to the extended focus on North American and Australian data present in this paper future studies could explore ethnic and socioeconomic differences present in method of introduction to opioids.

### Strengths and Limitations

This systematic review has some clear strengths, with the most notable being the methodological strengths. Firstly, this is the first systematic review to our knowledge that compares the method of introduction to opioids and treatment outcomes in OUD patients while in MAT. We were able to conduct six different meta-analyses on illicit opioid use, cocaine use, alcohol use, cannabis use, benzodiazepine use, and injection drug use. We employed rigorous screening methods to ensure all possible studies were included. Additionally, we presented our findings in a qualitative and quantitative method. Despite having a small number of studies included, the heterogeneity of the meta-analyses was less than 40%.

As with most systematic reviews, ours is not without limitations. The first limitation is that we were not able to conduct adjusted analyses. Unfortunately, not all the studies adjusted for confounding variables, which necessitates a more cautious interpretation of the findings. It is also important to mention that the included studies are before 2018, which may limit the impact of findings on the current opioid climate. Also, the analysis conducted was focused on North American or Australian data (the most available data), which minimizes the generalizability of the findings. We were also unable to conduct any analysis to detect publication bias due to a paucity of included studies. There is a lack of research on examining treatment outcome differences by the method of introduction to opioids as well as limited data on novel opioids and fentanyl derivatives. There is a need to not only to continue to examine this association through additional primary studies, but to also to investigate whether the type of opioids initially prescribed has ramifications on the risk of subsequently developing OUD. Additionally, standard urine screens may not be able to detect novel opioid. However, regardless of being able to detect novel opioids, our results did find a significant association for illicit opioid use and method of introduction to opioids. This finding may be a moderate estimation of the association and the actual association may be greater.

## Conclusion

This review highlights the differences found in illicit opioid use, cocaine use, alcohol use, injection drug use, and cannabis use in found in the cohort of patients that were introduced to opioids through a legitimate prescription and those introduced to opioids by recreational means. These differences are important for health policy makers and can help shape the success of these patients through further investigation.

## Data Availability Statement

All datasets presented in this study are included in the article/**Supplementary Material**.

## Author Contributions

NSa: contributed to the conception and design of the study, search strategy, screening and data extraction, analysis of results manuscript writing, and final review of the manuscript. MBh contributed to the conception and design of the study, screening and data extraction, analysis of results, and critical revision and final review. NSi, BP, AD’E, MT, HS, AH, NB-M, VR, DS, MB, EL: contributed to the methodological design, manuscript writing, critical revision, and final review of the manuscript. RD, MS, LT: contributed to the methodological design, critical revision, and final review of the manuscript. SS: contributed to the development of the search strategy and final review of the manuscript. ZS: contributed to the conception and design of the study, critical revision, and final approval of the manuscript. All authors contributed to the article and approved the submitted version.

## Funding

This research received no specific grant from any funding agency in the public, commercial or not-for-profit sectors. ZS is supported by grants from CIHR Award #156306, Bridge CIHR Sponsor Award #PJT-153429 and HAHSO Sponsor Award #HAH-16-04.

## Conflict of Interest

The authors declare that the research was conducted in the absence of any commercial or financial relationships that could be construed as a potential conflict of interest.
