# Supplementary material for: Treatment Outcomes in Patients With Opioid Use Disorder Who Were First Introduced to Opioids by Prescription: A Systematic Review and Meta-Analysis
Source: Front Psychiatry. 2020 Aug 28;11:812. doi: 10.3389/fpsyt.2020.00812 (PMC7485127; doi:10.3389/fpsyt.2020.00812)
Supplement: Supplementary file 2 [file Presentation_1.pdf]

Table 1. Search Strategy

MEDLINE	1 exp Analgesics, Opioid/<br>2 (opiate* or opioid* or fentanyl or narcotic* or dilaudid or oxycontin* or oxycod*).ti,ab.<br>3 1 or 2<br>4 exp Drug Prescriptions/<br>5 (prescript* or prescrib* or pharmaceutical* or legal*).ti,ab.<br>6 4 or 5<br>7 3 and 6<br>8 ((prescript* or prescrib* or pharmaceutical*) adj2 (opioid* or opiate* or dilaudid or fentanyl or codeine or oxyco*)).ti,ab.<br>9 7 or 8<br>10 Opioid-Related Disorders/<br>11 Heroin Dependence/<br>12 Substance-Related Disorders/<br>13 Substance Abuse, Intravenous/<br>14 ((opiate* or opioid* or heroin* or oxyco* or codeine* or dilaudid or fentanyl or drug* or substance*) adj2 (use* or using or misuse* or abus* or dependence* or dependent* or addict*)).ti,ab.<br>15 10 or 11 or 12 or 13 or 14<br>16 9 and 15<br>17 exp animals/ not (humans/ and exp animals/)<br>18 16 not 17<br>19 9 and 15
EMBASE	1 exp heroin dependence/<br>2 opiate/<br>3 exp opiate addiction/<br>4 substance abuse/<br>5 ((opiate* or opioid* or heroin* or oxyco* or codeine* or dilaudid or fentanyl or drug* or substance*) adj2 (use* or using or misuse* or abus* or dependence* or dependent* or addict*)).ti,ab.<br>6 1 or 2 or 3 or 4 or 5<br>7 ((prescript* or prescrib* or pharmaceutical*) adj2 (opioid* or opiate*

	or dilaudid or fentanyl or codeine or oxyco*)).ti,ab.<br>8 (prescript* or prescrib* or pharmaceutical* or legal*).ti,ab.<br>9 exp prescription/<br>10 exp prescription drug/<br>11 (opiate* or opioid* or fentanyl or narcotic* or dilaudid or oxycontin* or oxycod*).ti,ab.<br>12 exp narcotic analgesic agent/<br>13 11 or 12<br>14 8 or 9 or 10<br>15 13 and 14<br>16 7 or 15<br>17 6 and 16<br>18 limit 17 to human
PsycINFO	1 exp Opiates/<br>2 (opiate* or opioid* or fentanyl or narcotic* or dilaudid or oxycontin* or oxycod*).ti,ab.<br>3 exp Prescription Drugs/<br>4 1 or 2<br>5 (prescript* or prescrib* or pharmaceutical* or legal*).ti,ab.<br>6 3 or 5<br>7 4 and 6<br>8 ((prescript* or prescrib* or pharmaceutical*) adj2 (opioid* or opiate* or dilaudid or fentanyl or codeine or oxyco*)).ti,ab.<br>9 7 or 8<br>10 exp Heroin Addiction/ or exp Heroin/<br>11 exp Intravenous Drug Usage/<br>12 ((opiate* or oxyco* or opioid* or heroin* or codeine* or dilaudid or fentanyl or drug* or substance*) adj2 (use* or using or misuse* or abus* or dependence* or dependent* or addict*)).ti,ab.<br>13 10 or 11 or 12<br>14 9 and 13
	1 (MH "Drugs, Non-Prescription") OR (MH "Drugs, Prescription") OR (MH "Prescriptions, Drug") OR (MH "Drugs, Off-Label")

CINAHL	2 (MH "Substance Use Disorders")<br>3 (MH "Heroin") OR (MH "Substance Dependence")<br>4 (MH "Substance Abuse, Intravenous")<br>5 ((opiate* or opioid* or oxyco* or heroin* or codeine* or dilaudid or fentanyl or drug* or substance*) N2 (use* or using or misuse* or abus* or dependence* or dependent* or addict*))<br>6 (MH "Analgesics, Opioid")<br>7 (opiate* or opioid* or fentanyl or narcotic* or dilaudid or oxycontin* or oxycod*)<br>8 2 OR 3 OR 4 OR 5<br>9 ((prescript* or prescrib* or pharmaceutical*) n2 (opioid* or opiate* or dilaudid or fentanyl or codeine or oxyco*))<br>10 6 OR 7<br>11 (prescript* or prescrib* or pharmaceutical* or legal*)<br>12 1 OR 11<br>13 12 AND 13<br>14 9 OR 14<br>15 8 AND 15 (limiters- human)

Cooper 2018

Study Identification	Author Judgment	Justification
Appropriate Source Population	Low risk	Consecutive sample from a population representative of the condition under study
Sufficient Power/Sample Size	Low Risk	Modest sample size (N = 108)
Adjust for Confounders or Other Variables	Low Risk	Covariates included age, gender and other variables significant at the bivariate level.
Appropriate Statistical Analyses	Low Risk	Reported use of appropriate statistical analysis as required
Incomplete Outcome Data	Low to Moderate Risk	Multiple imputation with fully conditional specification to impute missing item-level data to maintain power for analysis
Outcome Measurement	Low Risk	Provided a detailed description of the outcome measures which are appropriate for the outcome of interest
Objective assessment of the outcome of interest	Low to Moderate Risk	Relied on self-report as the primary method to discern outcome status

Sanger 2018

Study Identification	Author Judgment	Justification
Appropriate Source Population	Low Risk	Consecutive sample from a population representative of the condition under study
Sufficient Power/Sample Size	Low Risk	Large sample size (N = 976)
Adjust for Confounders or Other Variables	Low Risk	Covariates included age, sex, methadone dose, treatment duration among others
Appropriate Statistical Analyses	Low Risk	Reported use of appropriate statistical analysis as required

Incomplete Outcome Data	Low Risk	Participants missing data were not included in analysis
Outcome Measurement	Low Risk	Provided a detailed description of the outcome measures which are appropriate for the outcome of interest
Objective assessment of the outcome of interest	Low to Moderate Risk	Relied on self-report as the primary method to discern outcome status

Canfield 2010

Study Identification	Author Judgment	Justification
Appropriate Source Population	Low Risk	Consecutive sample from a population representative of the condition under study
Sufficient Power/Sample Size	Low Risk	Small sample size (N = 75); provided power analysis
Adjust for Confounders or Other Variables	High Risk	Did not adjust for confounders; performed student t-tests
Appropriate Statistical Analyses	Low Risk	Reported use of appropriate statistical analysis as required
Incomplete Outcome Data	Unclear Risk	No information provided
Outcome Measurement	Low Risk	Provided a detailed description of the outcome measures which are appropriate for the outcome of interest
Objective assessment of the outcome of interest	Low to Moderate Risk	Relied on self-report as the primary method to discern outcome status

Tsui 2010

Study Identification	Author Judgment	Justification

Appropriate Source Population	Low Risk	Consecutive sample from a population representative of the condition under study
Sufficient Power/Sample Size	Low Risk	Modest sample size (N = 140)
Adjust for Confounders or Other Variables	Low Risk	Covariates included sociodemographics, depression, pain, current and prior substance use.
Appropriate Statistical Analyses	Low Risk	Reported use of appropriate statistical analysis as required
Incomplete Outcome Data	Low Risk	Missing data on the question which asked whether a physician had introduced them to opiates and were excluded from the analyses
Outcome Measurement	Low Risk	Provided a detailed description of the outcome measures which are appropriate for the outcome of interest
Objective assessment of the outcome of interest	Low to Moderate Risk	Relied on self-report as the primary method to discern outcome status

Dreifuss 2013

Study Identification	Author Judgment	Justification
Appropriate Source Population	Low Risk	Consecutive sample from a population representative of the condition under study
Sufficient Power/Sample Size	Low Risk	Large sample size (N = 360)
Adjust for Confounders or Other Variables	Unclear Risk	Unclear Information Provided
Appropriate Statistical Analyses	Low Risk	Reported use of appropriate statistical analysis as required
Incomplete Outcome Data	Unclear Risk	No information provided

Outcome Measurement	Low Risk	Provided a detailed description of the outcome measures which are appropriate for the outcome of interest
Objective assessment of the outcome of interest	Low to Moderate Risk	Relied on self-report as the primary method to discern outcome status
